# Enhancing the Techno-Functional Properties of Lentil Protein Isolate Dispersions Using In-Line High-Shear Rotor-Stator Mixing

**DOI:** 10.3390/foods13020283

**Published:** 2024-01-16

**Authors:** Nicolas Malterre, Francesca Bot, Emilie Lerda, Elke K. Arendt, Emanuele Zannini, James A. O’Mahony

**Affiliations:** 1 School of Food and Nutritional Sciences, University College Cork, T12 Y337 Cork, Ireland; nicolas.malterre@ucc.ie (N.M.); emilielerda@gmail.com (E.L.);; 2 Department of Food and Drug, University of Parma, 43124 Parma, Italy; francesca.bot@unipr.it; 3 APC Microbiome Institute Ireland, University College Cork, T12 Y337 Cork, Ireland; 4 Department of Environmental Biology, “Sapienza” University of Rome, 00185 Rome, Italy

**Keywords:** lentil proteins, plant proteins, in-line high-shear mixer, solubility, functional properties

## Abstract

In response to global challenges such as climate change and food insecurity, plant proteins have gained interest. Among these, lentils have emerged as a promising source of proteins due to their good nutritional profile and sustainability considerations. However, their widespread use in food products has been impeded by limited solubility. This study aimed to investigate the potential of high-shear mixing, a resource-efficient technique, to enhance lentil protein solubility and its functional properties. Red lentil protein isolate powders were rehydrated and subjected to a semi-continuous in-line high-shear treatment at 10,200 rpm for a timespan ranging from 0 to 15 min. The results highlighted a significant (*p* < 0.05) increase in solubility from 46.87 to 68.42% after 15 min of shearing and a reduction in particle size as a result of the intense shearing and disruption provided by the rotor and forced passage through the perforations of the stator. The volume-weighted mean diameter decreased from 5.13 to 1.72 µm after 15 min of shearing, also highlighted by the confocal micrographs which confirmed the breakdown of larger particles into smaller and more uniform particles. Rheological analysis indicated consistent Newtonian behaviour across all dispersions, with apparent viscosities ranging from 1.69 to 1.78 mPa.s. Surface hydrophobicity increased significantly (*p* < 0.05), from 830 to 1245, indicating exposure of otherwise buried hydrophobic groups. Furthermore, colloidal stability of the dispersion was improved, with separation rates decreasing from 71.23 to 24.16%·h^−1^. The significant enhancements in solubility, particle size reduction, and colloidal stability, highlight the potential of in-line high-shear mixing in improving the functional properties of lentil protein isolates for formulating sustainable food products with enhanced techno-functional properties.

## 1. Introduction

The need to tackle global challenges, ranging from climate change to food insecurity and chronic diseases, as well as the growing demand for healthy and sustainable food protein ingredients have recently driven interest in plant proteins [1,2,3]. Multiple protein sources including legumes, pseudocereals, cereals, tubers, grains, nuts, and seeds offer diverse nutritional profiles and health benefits [3,4]. Among these, pulses and more particularly lentils emerge as an interesting animal protein alternative, as they are rich in essential amino acids, dietary fibres, vitamins, and minerals as well as being affordable, sustainable and abundant raw materials [5]. Despite their positive attributes, the widespread utilisation of lentil proteins in food products has been impeded by their limited solubility [5,6]. Indeed, solubility is a fundamental functional property of proteins that plays a crucial role in processes such as gelation, foaming and emulsification where soluble proteins allow for the stabilisation of oil globules by adsorption at the oil/water interface [7,8,9]. On the other hand, the low solubility of protein-based ingredients, impacted by extraction processing parameters (e.g., pH changes, drying temperatures), can lead to processing challenges such as clumping, phase separation, sedimentation, and overall poor physical stability [6,10]. To enhance the functional properties of plant-based proteins, a range of strategies have been investigated, including chemical and enzymatic modifications, as well as physical treatments [11,12]. High-pressure homogenisation (HPH), a well-regarded technique known for its ability to modify protein structures and enhance their functional properties, has been widely used in recent years [13,14,15,16]. Several studies have shown that HPH could be used to improve the functional properties of plant-protein ingredients, particularly their solubility. More specifically, HPH treatment in the range of 70–150 MPa could lead to a 20 to 30% increase in solubility [17,18,19]. Similarly, Saricaoglu (2020) [20] has shown that HPH treatment in the pressure range of 0–150 MPa could increase the solubility of the protein ingredients from 32 to 46%. Other studies have investigated the effect of HPH on protein ingredients for the formulation of lentil protein stabilised emulsions, such as Jeske et al. (2019) [21] and Primozic et al. (2018) [22], who studied the suitability of HPH (34 and 103 MPa) to improve lentil protein isolate (LPI) for use in nanoemulsions. The results showed that HPH treatment allowed for a reduction in particle size and hydrophobicity. However, while effective, HPH comes with challenges as it is resource-intensive, both in terms of capital cost and energy consumption [23,24].

On the other hand, high-shear mixers, which are composed of a rotor-stator allowing for a shearing rate in the range of 20,000–100,000 s^−1^, are highly efficient in increasing powder solubility in a cost-effective manner [25,26]. This physical treatment has been used extensively in the dairy industry during batch compounding for the disintegration and integration of powder particles into various aqueous phases. Indeed, O’Sullivan et al. (2017) [27] compared different methods of powder rehydration and found that the use of an in-line high-shear mixer allowed for the best reduction in particle size of the dispersion from 20 to 10 µm. Furthermore, extensive literature is available on the use of in-line shear mixers for the formulation of stable emulsions [28,29,30,31]. Scholz and Keck (2015) [32] have shown that an in-line high-shear rotor-stator mixer could allow for the formulation of stable emulsions with droplet size slightly larger than the one obtained by high-pressure homogenization; however, no evidence of differences in the functionalities of the emulsions were found.

Even though the use of in-line high-shear mixers in the dairy industry for the disruption of dairy powder particles and formulation of stable emulsions has been well documented, to the author’s knowledge, there is no information available on the influence of in-line high-shear mixers on plant protein solubility and functional properties. Consequently, this study aims to investigate the potential of high-shear mixing to enhance the functional properties of lentil protein dispersions. The results of this study will support the development of cost-effective, sustainable processing solutions for the use of plant-based protein ingredients in food systems including infant formulae, milk, and cheese alternatives.

## 2. Materials and Methods

### 2.1. Preparation of Lentil Protein Isolate Dispersions

The lentil protein dispersions were prepared by rehydrating red lentil protein isolate powder (72.15% protein, *w*/*w*), provided by Döhler GmbH (Darmstadt, Germany), at a concentration of 5% powder (*w*/*v*) (corresponding to 3.61% protein (*w*/*v*)) in pre-heated deionised water (70 °C), stirred magnetically at 300 rpm for 1 h at 20 °C. While the protein content of the lentil protein isolate powder was not as high as existing protein isolates generally used in the formulation of infant nutritional products, it is in line with previous studies on plant-based nutritional products [21,33,34]. The main non-protein constituents of such ingredients are typically fat, starch and fibre, with the starch and fibre components, in particular, having the potential to contribute to functional properties (see Section 3.2 for further details). The dispersions were then adjusted to pH 6.8 [33,35] and allowed to rehydrate fully at 4 °C for 18 h, after which the samples were equilibrated at 20 °C and pH was readjusted, if necessary, to pH 6.8. This pH was chosen to align with that typical of infant formula as employed in previous research from our group [33].

### 2.2. High-Shear Mixing Treatment

The lentil protein dispersion was subjected to a semi-continuous in-line high-shear treatment using a Silverson laboratory high-shear mixer (L5M-A; Silverson, East Longmeadow, MA, USA), equipped with an in-line mixing assembly to allow for recirculation. A peristaltic pump (Watson Marlow, Marlow, UK) was used to feed the shearing head. A valve was set on the outlet of the shearing head to apply 0.5 bar of back pressure and the outlet was directed to the feeding tank. Samples were collected from the outlet after 0 (no shearing), 1, 3, 5 and 15 min of shearing at 10,200 rpm. A control sample, which did not pass through the shearing system was included in the analysis.

### 2.3. Particle Size Distribution

A Mastersizer 3000 (Malvern Instruments, Worcestershire, UK) static light scattering device fitted with an automated Hydro MV wet and dry cell disperser was used to evaluate the particle size distribution (PSD) of the dispersion and powder sample forms respectively. The protein’s refractive index was set at 1.338, the dispersant’s refractive index at 1.33, and the adsorption index was 0.001. The dispersions were diluted in ultrapure water until 10% laser obscuration was achieved (at 20 °C). The volume-weighted mean particle size (D[4,3]), specific surface area, and the particle sizes below which 10 and 90% of the sample volume are detected (Dv(10) and Dv(90)) are presented as the results.

### 2.4. Confocal Laser Scanning Microscopy

Microstructural analysis of the dispersions was performed using an OLYMPUS FV1000 confocal laser scanning biological microscope (Olympus Corporation, Tokyo, Japan). The dispersions were prepared as described by Malterre et al. (2023) [34] with the following modifications, whereby 1 mL of the dispersion was mixed in 4 mL of low gelling temperature agarose (Sigma-Aldrich, St. Louis, MO, USA) solution (1.5%, *w*/*v*) at 37 °C to fix the dispersion and allow for higher quality imaging. As described by Grasso et al. (2021) [36], 20 µL of Fast Green FCF aqueous solution (200 μL of 0.1 g/L) was added to 1 mL of the dispersion-agarose sample, which was then incubated at 20 °C until gelation. Fast Green FCF was excited at 633 nm, and representative images, performed using a 20× objective lens, were reported whereby protein was stained red.

### 2.5. Rheological Analysis

A modular compact rheometer MCR 102e (Anton Paar, Graz, Austria) fitted with a concentric cylinder geometry was used to assess the rheological characteristics of the dispersions. The rotor’s diameter was 26.7 mm, the cell’s internal diameter was 28.9 mm, and the distance between the two parts at the geometrical base was 2 mm. After loading the sample (20 mL) into the cylinder and preheating it to 20 °C, the shear rate was increased to 100 s^−1^ over 30 s and maintained for 30 s. A constant temperature of 20 °C was applied throughout the measurement, and viscosity was continuously recorded. The Ostwald-de Waele model (Equation (1)) was used to describe the rheological properties of the protein dispersions while ensuring that R^2^ ≥ 0.95.

τ = Kγ̇^n^ (1)

where τ is the shear stress (mPa), γ̇ is the shear rate (s^−1^), K is the flow consistency coefficient (mPa.s^n^) and n is the flow behaviour index (dimensionless).

### 2.6. Protein Solubility

An RC5C plus centrifuge (Thermo ScientificTM SorvallTM, Waltham, MA, USA) with a GSA rotor was used to determine the protein solubility of the dispersions [33]. The protein concentration in the supernatant of the dispersions, obtained after centrifugation at 1000× *g* for 20 min at 20 °C, was measured using the Kjeldahl method, with a nitrogen-to-protein conversion factor of 6.25. The results are expressed as a percentage of protein in the supernatant (soluble protein) as compared to the total protein content of the dispersions (3.61% (*w*/*v*)).

### 2.7. Protein Profile Analysis

The protein profile of the dispersions, and their fractions, were assessed using sodium dodecyl sulphate-polyacrylamide gel electrophoresis (SDS-PAGE) with precast gels (Mini-PROTEAN TGX, Bio-Rad Laboratories, Hercules, CA, USA), following the method developed by Laemmli (1970) [37], with minor modifications. The samples were centrifugated at 1000× *g* for 20 min at 20 °C using an RC5C plus centrifuge (Thermo Scientific™ Sorvall™, Waltham, MA, USA) with a GSA rotor to isolate the soluble protein in the supernatant and diluted to a protein concentration of 2 mg/mL. In method I (non-reducing conditions), 100 µL of the diluted supernatant was mixed with 100 µL of sample loading buffer (2× Laemmli sample buffer, Bio-Rad Laboratories, Hercules, CA, USA) whereas, in method II (reducing conditions), 100 µL of the diluted dispersion was mixed with 95 µL of sample loading buffer and 5 µL of β-mercaptoethanol. Each well was loaded with 7 μL of sample solution and 5 µL of SDS-PAGE broad range protein standard (Bio-Rad laboratories, Hercules, CA, USA) was loaded as a protein molecular weight standard. The gels were run in a running buffer (10× Tris/Glycine/SDS, Bio-Rad Laboratories, Hercules, CA, USA) at 160 V for 1 h, then fixed for 15 min using a solution of water:methanol:acetic acid (40:50:10), stained with Coomassie Brilliant Blue R-250 (Bio-Rad Laboratories, Hercules, CA, USA) and destained using a solution of water:methanol:acetic acid (50:40:10) until a clear background was achieved. The gels were then stored at 4 °C in a 5% (*v*/*v*) acetic acid solution overnight to reverse the shrinkage induced during the fixing and destaining steps.

### 2.8. Surface Hydrophobicity

Hydrophobicity of proteins in the supernatant (dispersion centrifugated at 1000× *g* for 20 min as described in Section 2.6) was measured using the method previously described by Nakai (2003) [38] and Goulding et al. (2021) [39], with minor modifications, using the hydrophobic probe 8-Anilino-1-naphthalenesulfonic acid (ANS). Samples were diluted to each of the following concentrations, 0.003, 0.006, 0.009, 0.012, and 0.015% (*v*/*v*) in sample buffer (0.01 M phosphate buffer, pH 7) and 100 µL of the diluted solutions were transferred to a black 96 microwell plate, after which 50 µL of ANS solution (8 mM in 0.1 M phosphate buffer) was added. The well plate was then covered with foil and gently rocked for 15 min on a plate shaker. After this, the fluorescence intensity of the samples was measured using a Varioskan Flash microplate reader (Fisher Scientific, Ireland) with an excitation wavelength of 390 nm and an emission wavelength of 470 nm. The fluorescence intensity was plotted as a function of protein concentration (%, *v*/*v*) and only linear regressions with an R^2^ higher than 0.98 were considered. The slope (S0) value was used to compare the surface hydrophobicity of proteins in the different samples.

### 2.9. Accelerated Physical Stability

The colloidal stability of the dispersions was measured using an analytical centrifuge (LUMiSizer^®^, LUM GmbH, Berlin, Germany). The samples were centrifugated through two successive cycles at 800 and 3000 rpm for 10 min each, with near-infrared light illuminating the samples throughout the analysis and the intensity of the transmitted light over the entire length of the sample was measured every 10 s. The separation rate was determined by plotting the integral of the intensity of the transmitted light over time and extracting the slope (%.h^−1^). The light transmission profiles before centrifugation, after the first cycle and after the second cycle were extracted and plotted.

### 2.10. Statistical Data Analysis

All samples were formulated in independent triplicate trials and all analyses were conducted in triplicate, unless otherwise stated. The data generated was subject to one-way analysis of variance (ANOVA) using R version 4.3.1 (R foundation for statistical computing, Vienna, Austria). A paired comparison test was used to determine statistically significant differences (*p* < 0.05) between mean values for different samples, at a 95% confidence level.

## 3. Results and Discussion

### 3.1. Particle Size Distribution and Microstructure

The particle size distribution (PSD) parameter, volume-weighted mean particle diameter (D[4,3]) of the lentil powders, control sample and that processed through the in-line high-shear mixer are shown in Table 1. The red lentil protein isolate powder ingredient displayed a D[4,3] value of 30.7 ± 0.06 µm in line with the literature [34,40,41]. The control red lentil protein dispersion and the sample processed through the in-line high-shear mixer for 0 min displayed similar (*p* > 0.05) D[4,3] values, of 5.13 ± 0.58 µm and 5.00 ± 0.04 µm, respectively, indicating that the pumping through the semi-continuous system and application of back pressure did not affect the particle size distribution. Interestingly, increasing the shearing time caused a significant (*p* < 0.05) decrease in the PSD parameters, with a decrement in D[4,3] to 2.81 ± 0.06, 2.17 ± 0.04 and 1.72 ± 0.01 µm after 1, 5 and 15 min of processing, respectively. This progressive reduction in particle size can be attributed to the high shearing forces induced by the rotor of the mixing head, which causes the breakage of the powder particles with the forced passage of the particles through the perforations of the stator [30,42,43].

**Figure 1:**
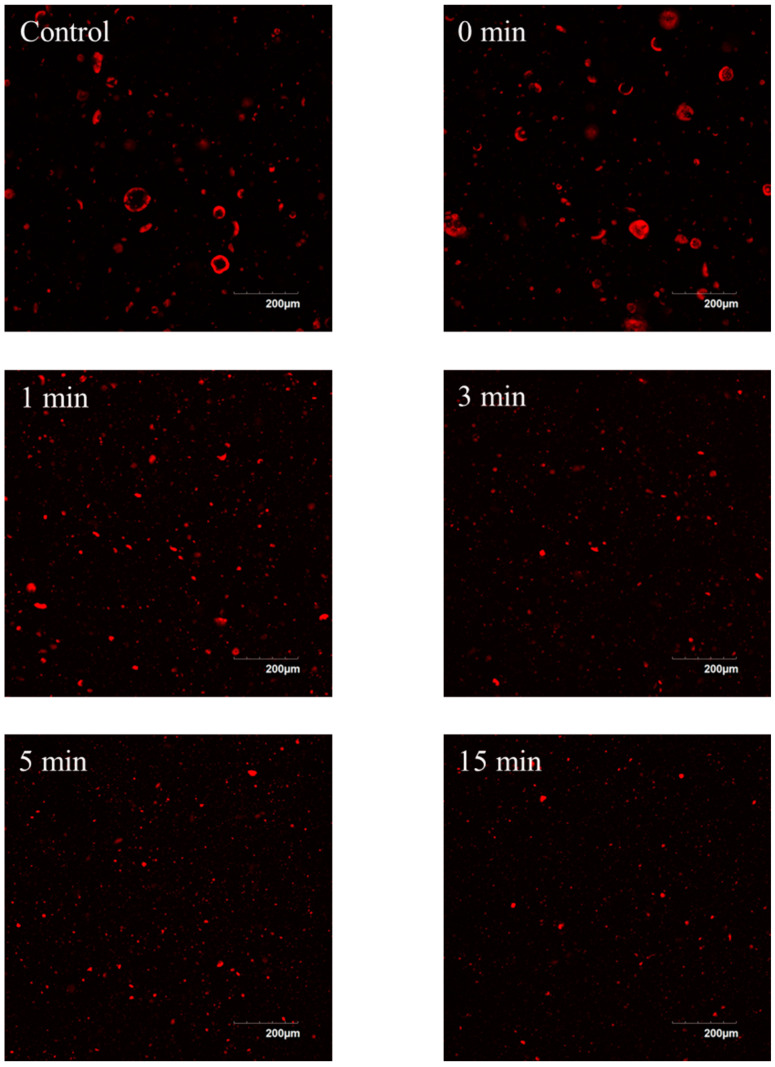
Confocal laser micrographs of lentil protein isolate dispersions (5% powder, *w*/*v*) of untreated (control) and treated with high-shear mixing for 0, 1, 3, 5, and 15 min.

Changes in particle size are also visible on the confocal micrographs (Figure 1), as the control and 0 min dispersions displayed large particles in the range of 40–50 µm. Furthermore, micrographs of the 1 min dispersion, showed heterogeneous particles in the range of 1–5 µm and 15–25 µm. Longer processing up to 15 min showed further reductions of the population of large particles and an increase in small particles as illustrated by an increase in the intensity of red particles on the micrographs (Figure 1).

**Figure 2:**
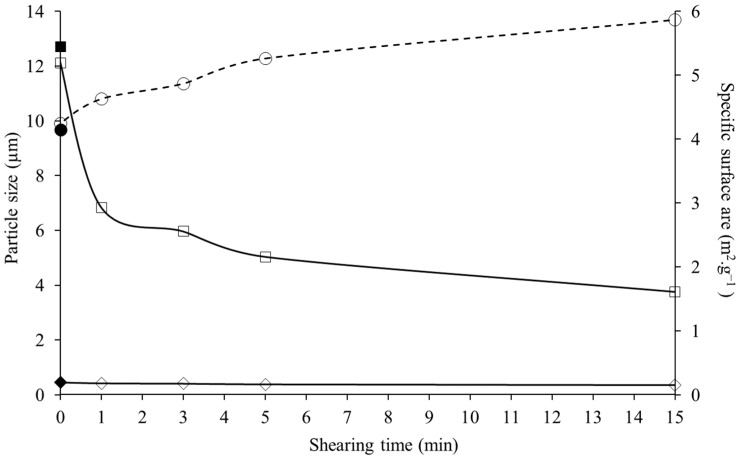
Particle size distribution parameters (-◇-, Dv(10), particle size below which 10% of sample volume is found), (-□- Dv(90), particle size below which 90% of sample volume is found), and (--○--, specific surface area) of protein dispersions (5% powder *w*/*v*) untreated (control) and treated with high-shear for 0, 1, 3, 5, and 15 min. Filled shapes (-■-, -◆- and -●-) correspond to the control dispersion.

The reduction in number of bigger particles can also be shown through the particle size parameters D(90) (particle size below which 90% of sample volume is found), which progressively decreased throughout the process from 12.71 ± 3.33 µm for the control dispersion to 6.83 ± 0.35 and 3.76 ± 0.07 µm after 1 and 15 min of shearing (Figure 2). On the other hand, the D(10), which represents the particle size below which 10% of the sample volume is found, had minimal reduction (*p* > 0.05) through shearing from 0.46 ± 0.05 µm for the control dispersion and 0.42 ± 0.01 µm for the dispersion treated for 1 min. This parameter then plateaued (*p* > 0.05) with increasing shearing time to reach a D(10) value of 0.35 ± 0.01 µm after 1 min. Furthermore, the particle disruption induced by the high-shearing caused a significant (*p* < 0.05) increase in specific surface area from 4.14 ± 0.46 m^2^·g^−1^ for the control dispersion to 5.87 ± 10 m^2^·g^−1^ after 15 min of shearing (Figure 2). Higher specific surface area could allow for greater exposure of each particle to water and possibly better dissolution of the powder as demonstrated by the Noyes Whitney equation, which details a link between the exposed surface and the dissolution rate of the powder [44,45]. The results are in agreement with the literature as similar results on the effect of high-shear mixing on particle disruption have been found in the literature for powders. In particular, Pacek et al. (2007) [42] and Padron and Özcan-Taskin (2018) [46], found that an in-line high-shear mixer at 8000 rpm, caused the reduction of particles of silicon from 90 µm to 9 µm after 20 min and from 100 µm to 15 µm after 30 min of shearing. The authors have also shown that in the timespan used in the present study (<15 min), in-line high-shearing did not allow for the disruption of aggregates below 1–2 µm. Similarly, O’Sullivan et al. (2017) [27] have found that the use of in-line high-shear mixer for the rehydration and dispersion of dairy powders (skim milk powder and milk protein isolate) at a concentration of 7.2% (*w*/*w*) caused a significant reduction of particle size from 100 to 20 µm after 15 min, while the size of the smaller particles plateaued in the range 0.1–1 µm. Overall, these results show that in-line high-shear mixing has the potential to effectively reduce the particle size of lentil protein isolates across both large and small particles/aggregates, which could have potential benefit in increasing the dissolution rate and improving overall rehydration.

### 3.2. Rheological Properties

The apparent viscosity of the dispersions as well as their flow properties n (-) and k (mPa.s^n^) as defined by fitting the Ostwald de Waele model, are provided in Table 2. The apparent viscosity at 100 s^−1^ and 20 °C ranged between 1.69 ± 0.3 and 1.78 ± 0.34 mPa.s with no significant differences (*p* > 0.05) between the dispersions. Similar apparent viscosity values were measured for lentil protein dispersions with protein concentrations of 4% (*w*/*w*) [20]. The Ostwald de Waele model allowed for the accurate description of the rheological properties of the dispersions (R^2^ > 0.98), and similar (*p* > 0.05) consistency coefficient ranging between 1.57 × 10^−3^ ± 0.81 × 10^−3^ and 1.87 × 10^−3^ ± 0.72 × 10^−3^ mPa.s were observed over the range of treatments. The flow behaviour index of all dispersions ranged between 1.00 ± 0.06 and 1.03 ± 0.09 mPa.s^n^, indicating Newtonian behaviour of the dispersions. Similar results have been observed by Jeske et al. (2019) [21] who studied the HPH treatment of lentil protein isolate emulsions at a concentration of 3% (*w*/*w*). Furthermore, the impact of starch and fibre content on the rheological properties of plant protein emulsions was not evaluated as part of this study. According to Dikeman and Fahey (2006) [47] changes in starch or fibre content could modify the viscosity of the samples and therefore have an impact on the shearing and mechanical forces induced by high-shear mixing; therefore, future studies should investigate the impact of starch and fibre content on the techno-functional properties of lentil protein isolate dispersions for use in food systems.

### 3.3. Protein Solubility

Protein solubility is an essential functional property which strongly influences other protein functional properties, such as emulsifying, foaming or gelling properties [7,9]. The control dispersion had a protein solubility of 46.87 ± 1.55%, similar (*p* > 0.05) to the dispersion treated for 0 min displaying a solubility of 48.04 ± 4.03% (Table 1), as expected for plant protein ingredients which have low solubility as compared to dairy isolates (>90% in similar conditions) [6]. The high shearing generated through the treatment allowed for a 20% increase in solubility when treated for 1 min (57.18 ± 5.65%), and continuously increased up to a value of 68.42 ± 8.04% for the dispersion treated for 15 min, allowing for 42% increase in solubility. The increase in solubility can be attributed to the particle breakage caused by the intense shearing as observed in Table 2. Similar results have been observed by other authors who showed that mechanical shearing up to 8000 rpm can increase the solubility of protein ingredients by breaking down the protein aggregates and facilitating their dispersion and dissolution [27,48].

The breakages and disruptions induced by the in-line high-shear treatment, reducing the particle size, could have allowed for better dispersion of the agglomerated powder particles Schuck et al. (2012) [49], increasing the rehydration and solubility of the ingredients as shown by other authors who worked on the improvement of protein ingredients through mechanical processing such as the well-documented use of HPH for the improvement of plant protein functionalities [18,50,51,52,53,54].

**Figure 3:**
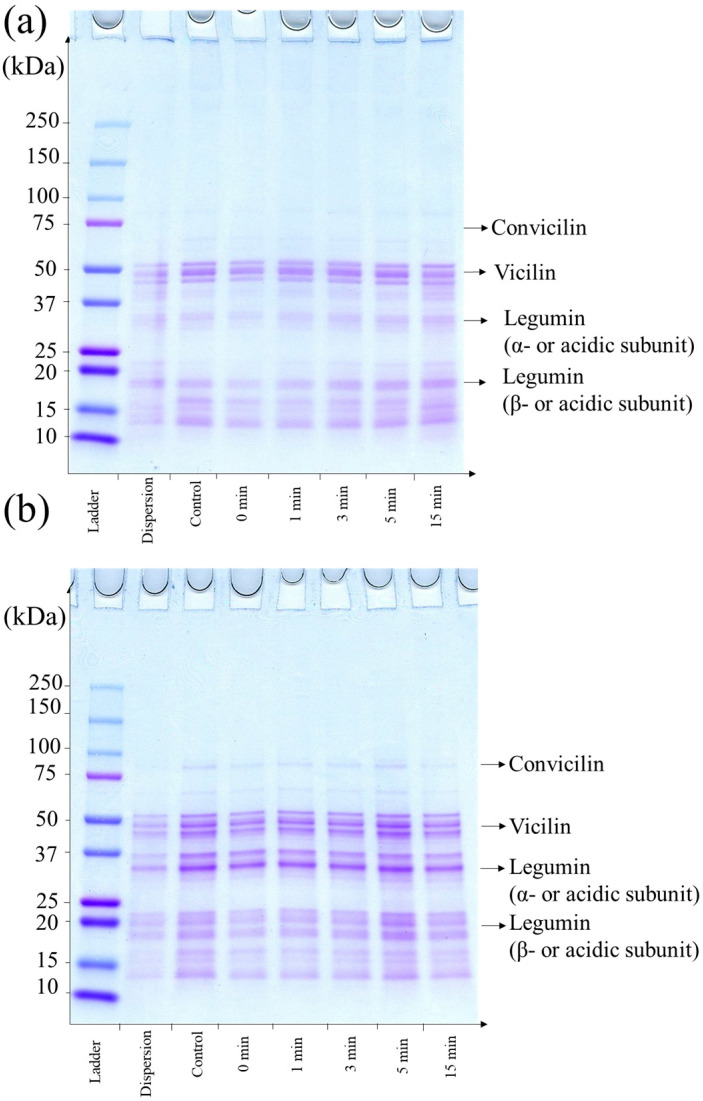
Representative sodium dodecyl sulphate-polyacrylamide gel electrophoretograms (SDS-PAGE) of lentil protein isolate dispersions (5% *w*/*v*) untreated, control supernatant untreated and supernatant treated with high-shear-mixing for 0, 1, 3, 5 and 15 min under non reducing (**a**) and reducing (**b**) conditions.

A better understanding of the protein solubilisation as affected by processing time was obtained by analysing the protein profile of the soluble fractions of the dispersions (Figure 3a,b). The supernatant of lentil protein isolate dispersions had proteins with molecular weight of 50 kDa, which may correspond to vicilin subunits, and the bands at 37 and 25 kDa may correspond to the acidic and basic subunits of legumin. Under reducing conditions, legumin dissociated into its acidic and basic subunits due to the dissociation of disulphide bonds, resulting in a decrease in the intensity of the bands at 50 and 37 kDa and an increase in the intensity of the bands at 20–25 kDa. Similar results have been observed by Jarpa-Parra et al. (2015) [55], Barbana and Boye (2013) [56], Joshi et al. (2012) [57], and Alonso-Miravalles et al. (2019) [41] The protein profile of all the samples were similar to the one of the control sample, indicating that in-line high-shear treatment did not affect the profile of solubilised proteins.

### 3.4. Surface Hydrophobicity

Understanding hydrophobic interactions is crucial for the development of food products containing high concentrations of protein ingredients, as they have a strong contribution to stability and overall interfacial properties. The control sample had the lowest value of 830 ± 123, similar (*p* > 0.05) to the sample treated for 0 min, revealing a negligible effect of the pumping in the semi-continuous system studied (Table 1). Treating the sample at 10,200 rpm for 1 min allowed for a significant (*p* < 0.05) increase of the surface hydrophobicity to 1044 ± 26, which reached the maximum value of 1245 ± 43 for the sample treated for 5 min. This increase in surface hydrophobicity could be attributed to exposure of hydrophobic residues, otherwise buried, as a result of mechanical disruption [39,52]. The results agree with the literature, where the application of mechanical forces (i.e., shear, cavitation, turbulences) caused an increase in surface hydrophobicity and thus an improvement of plant protein functionalities (i.e., emulsifying properties) [52,58].

### 3.5. Colloidal Stability

**Figure 4:**
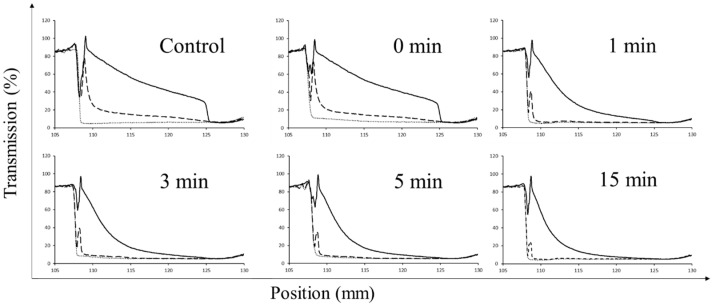
Accelerated physical stability profiles of lentil protein isolate dispersions (5% *w*/*v* powder) untreated (control) and treated with high-shear treatment for 0, 1, 3, 5 and 15 min. Original light transmission of the dispersions (time 0 min) (….) together with the profile after the first cycle (10 min at 800 rpm) (- -) and the profile after the second cycle (10 min at 3000 rpm) (-) are shown.

The physical stability of the dispersions was analysed by measuring the transmission of light through the samples during centrifugation. In Figure 4, representative light transmission profiles for each of the dispersions treated at 10,200 rpm from 0 to 15 min are shown. The control and the sample treated for 0 min showed low physical stabilities with separation rates of 71.23 ± 18.15%.h^−1^ and 69.67 ± 18.85%.h^−1^, after the second centrifugating cycle, respectively. In addition, at low centrifugation speed (i.e., 800 rpm), it can be observed that the treated dispersions (1, 3, 5 and 15 min) did not undergo any changes through the first cycle, indicating relative physical stability of the large, dispersed particle as also underlined by Crowley et al. (2019) [59]. When increasing the speed to 3000 rpm during the second cycle, complete sedimentation and destabilisation of the samples can be measured. In particular, the separation rate of the dispersion decreased sharply (*p* < 0.05) with the treatment of 1 min (36.57 ± 5.19%.h^−1^) indicating a slower movement of particles in the samples as a result of particle disruption and particle size reduction, which resulted in more colloidally stable dispersions. The separation rate decreased progressively to reach a value of 24.16 ± 2.58%.h^−1^ for the dispersion treated for 15 min. The results are in agreement with the literature, particularly Moll et al. (2021) [60], who found that the reduction in particle size caused by physical treatment with microfluidisation allowed for an increase in colloidal stability and Jeske et al. (2019) [21] using HPH at 18 MPa observed a reduction in separation rate from 48 to 22%.h^−1^.

## 4. Conclusions

The effect of in-line high-shear mixing at 10,200 rpm on the techno-functional properties of lentil protein isolate was studied during treatment times ranging between 0 and 15 min. It was shown that the high-shear and long time provided by the mixer allowed for a 42% improvement in solubility of the protein ingredients from 46.87 ± 1.55% of soluble protein at 0 min up to 68.42 ± 8.04% when treated for 15 min. This increase in solubility has been attributed to the high shearing induced at the rotor and the passage through the small perforations of the stator, with a concomitant significant (*p* < 0.05) reduction in particle size from 5.13 ± 0.58 µm (at 0 min) to 1.72 ± 0.01 µm after 15 min. Furthermore, the particle disruption caused an increase in hydrophobicity as hydrophobic groups exposed to the surface have been observed, which can be associated with an improvement of the emulsifying properties of the lentil protein isolate. The lentil protein isolate dispersions after in-line high-shear treatment had improved colloidal stability with values of 69.67%.h^−1^ at 0 min, down to 35.77%.h^−1^ after 1 min of treatment and further treatment for 15 min reduced it to 24.16%.h^−1^. These results show that in-line high-shear mixing has the potential to improve the functional properties of lentil protein isolate dispersions for the formulation of more sustainable food products, including infant formulae with enhanced techno-functional properties.

## Figures and Tables

**Table 1:** Particle size distribution parameters, protein solubility, surface hydrophobicity and separation rate for lentil protein isolate powder and protein dispersions (5% powder, *w*/*v*) of untreated (control) and treated with high-shear mixing for 0, 1, 3, 5 and 15 min.

	D[4,3] (µm)	Span (µm)	Protein Solubility (%)	Surface Hydrophobicity (-)	Separation Rate (%.h^−1^)
Powder	30.7 ± 0.06 ^a^	1.84 ± 0.02 ^a^	n/a	n/a	n/a
Control	5.13 ± 0.58 ^b^	7.25 ± 2.98 ^b^	46.87 ± 1.55 ^a^	830 ± 123 ^a^	71.23 ± 18.15 ^a^
0 min	5.00 ± 0.04 ^b^	6.96 ± 0.16 ^b^	48.04 ± 4.03 ^a^	852 ± 73 ^a^	69.67 ± 18.85 ^a^
1 min	2.81 ± 0.06 ^c^	4.28 ± 0.22 ^c^	57.18 ± 5.65 ^b^	1044 ± 26 ^b^	36.57 ± 5.19 ^b^
3 min	2.49 ± 0.18 ^d^	3.99 ± 0.22 ^c^	63.69 ± 7.14 ^c^	1116 ± 70 ^bc^	30.81 ± 2.63 ^bc^
5 min	2.17 ± 0.04 ^e^	3.72 ± 0.06 ^d^	63.94 ± 5.70 ^c^	1245 ± 43 ^c^	28.89 ± 3.01 ^cd^
15 min	1.72 ± 0.01 ^f^	3.14 ± 0.02 ^e^	68.42 ± 8.04 ^d^	1236 ± 41 ^c^	24.16 ± 2.58 ^d^

Values within a column that share a superscript are not significantly different from one another (*p* < 0.05). D[4,3] = volume-weighted mean particle diameter. Span = measurement of the width of the distribution calculated as (Dv(90) − Dv(10))/Dv(50). n/a: not applicable.

**Table 2:** Apparent viscosity (η at 100 s^−1^, 20 °C), flow behaviour (n) and consistency coefficient (K) of protein dispersions (5%, *w*/*v*, powder) untreated (control) and treated with high-shear treatment for 0, 1, 3, 5 and 15 min.

Treatment	η (mPa.s)	n (-)	K (mPa.s^n^)
Control	1.74 ± 0.35 ^a^	1.02 ± 0.06 ^a^	1.73 × 10^−3^ ± 0.78 × 10^−3 a^
0 min	1.75 ± 0.33 ^a^	1.02 ± 0.07 ^a^	1.77 × 10^−3^ ± 0.76 × 10^−3 a^
1 min	1.69 ± 0.3 ^a^	1.02 ± 0.07 ^a^	1.57 × 10^−3^ ± 0.81 × 10^−3 a^
3 min	1.73 ± 0.26 ^a^	1.00 ± 0.06 ^a^	1.70 × 10^−3^ ± 0.60 × 10^−3 a^
5 min	1.72 ± 0.32 ^a^	1.03 ± 0.09 ^a^	1.67 × 10^−3^ ± 0.75 × 10^−3 a^
15 min	1.78 ± 0.34 ^a^	1.00 ± 0.05 ^a^	1.87 × 10^−3^ ± 0.72 × 10^−3 a^

Values within a column that share a superscript are not significantly different from one another (*p* > 0.05).

## Data Availability

Data will be made available on request.

## References

[1] (2022). World Population Prospects 2022: Summary of Results.

[2] Henchion M., Hayes M., Mullen A., Fenelon M., Tiwari B. (2017). Future Protein Supply and Demand: Strategies and Factors Influencing a Sustainable Equilibrium. Foods.

[3] Day L., Cakebread J.A., Loveday S.M. (2022). Food Proteins from Animals and Plants: Differences in the Nutritional and Functional Properties. Trends Food Sci. Technol..

[4] Shaghaghian S., McClements D.J., Khalesi M., Garcia-Vaquero M., Mirzapour-Kouhdasht A. (2022). Digestibility and Bioavailability of Plant-Based Proteins Intended for Use in Meat Analogues: A Review. Trends Food Sci. Technol..

[5] Can Karaca A., Low N., Nickerson M. (2011). Emulsifying Properties of Chickpea, Faba Bean, Lentil and Pea Proteins Produced by Isoelectric Precipitation and Salt Extraction. Food Res. Int..

[6] Grossmann L., McClements D.J. (2023). Current Insights into Protein Solubility: A Review of Its Importance for Alternative Proteins. Food Hydrocoll..

[7] Zhao H., Shen C., Wu Z., Zhang Z., Xu C. (2020). Comparison of Wheat, Soybean, Rice, and Pea Protein Properties for Effective Applications in Food Products. J. Food Biochem..

[8] Ebert S., Gibis M., Terjung N., Weiss J. (2020). Survey of Aqueous Solubility, Appearance, and pH of Plant Protein Powders from Carbohydrate and Vegetable Oil Production. LWT.

[9] Day L. (2013). Proteins from Land Plants—Potential Resources for Human Nutrition and Food Security. Trends Food Sci. Technol..

[10] Grossmann L., Weiss J. (2021). Alternative Protein Sources as Technofunctional Food Ingredients. Annu. Rev. Food Sci. Technol..

[11] Bou R., Navarro-Vozmediano P., Domínguez R., López-Gómez M., Pinent M., Ribas-Agustí A., Benedito J.J., Lorenzo J.M., Terra X., García-Pérez J.V. (2022). Application of Emerging Technologies to Obtain Legume Protein Isolates with Improved Techno-Functional Properties and Health Effects. Compr. Rev. Food Sci. Food Saf..

[12] Mirmoghtadaie L., Shojaee Aliabadi S., Hosseini S.M. (2016). Recent Approaches in Physical Modification of Protein Functionality. Food Chem..

[13] Gharibzahedi S.M.T., El Kantar S., Pasdar N., Altintas Z., Koubaa M., Bhat Z.F., Morton J.D., Bekhit A.E.-D.A., Suleria H.A.R. (2023). Chapter 7—Emerging Technologies for Processing of Plant Proteins. Processing Technologies and Food Protein Digestion.

[14] Sá A.G.A., Laurindo J.B., Moreno Y.M.F., Carciofi B.A.M. (2022). Influence of Emerging Technologies on the Utilization of Plant Proteins. Front. Nutr..

[15] Avelar Z., Vicente A.A., Saraiva J.A., Rodrigues R.M. (2021). The Role of Emergent Processing Technologies in Tailoring Plant Protein Functionality: New Insights. Trends Food Sci. Technol..

[16] Tobin J., Heffernan S.P., Mulvihill D.M., Huppertz T., Kelly A.L., Datta N., Tomasula P.M. (2015). Applications of High-Pressure Homogenization and Microfluidization for Milk and Dairy Products. Emerging Dairy Processing Technologies.

[17] Keuleyan E., Gélébart P., Beaumal V., Kermarrec A., Ribourg-Birault L., Le Gall S., Meynier A., Riaublanc A., Berton-Carabin C. (2023). Pea and Lupin Protein Ingredients: New Insights into Endogenous Lipids and the Key Effect of High-Pressure Homogenization on Their Aqueous Suspensions. Food Hydrocoll..

[18] Zhao S., Huang Y., McClements D.J., Liu X., Wang P., Liu F. (2022). Improving Pea Protein Functionality by Combining High-Pressure Homogenization with an Ultrasound-Assisted Maillard Reaction. Food Hydrocoll..

[19] Moll P., Salminen H., Griesshaber E., Schmitt C., Weiss J. (2022). Homogenization Improves Foaming Properties of Insoluble Pea Proteins. J. Food Sci..

[20] Saricaoglu F.T. (2020). Application of High-Pressure Homogenization (HPH) to Modify Functional, Structural and Rheological Properties of Lentil (*Lens culinaris*) Proteins. Int. J. Biol. Macromol..

[21] Jeske S., Bez J., Arendt E.K., Zannini E. (2019). Formation, Stability, and Sensory Characteristics of a Lentil-Based Milk Substitute as Affected by Homogenisation and Pasteurisation. Eur. Food Res. Technol..

[22] Primozic M., Duchek A., Nickerson M., Ghosh S. (2018). Formation, Stability and in Vitro Digestibility of Nanoemulsions Stabilized by High-Pressure Homogenized Lentil Proteins Isolate. Food Hydrocoll..

[23] Fayaz G., Soleimanian Y., Mhamadi M., Turgeon S.L., Khalloufi S. (2022). The Applications of Conventional and Innovative Mechanical Technologies to Tailor Structural and Functional Features of Dietary Fibers from Plant Wastes: A Review. Compr. Rev. Food Sci. Food Saf..

[24] Calligaris S., Plazzotta S., Bot F., Grasselli S., Malchiodi A., Anese M. (2016). Nanoemulsion Preparation by Combining High Pressure Homogenization and High Power Ultrasound at Low Energy Densities. Food Res. Int..

[25] O’Sullivan J.J., Drapala K.P., Kelly A.L., O’Mahony J.A. (2018). The Use of Inline High-Shear Rotor-Stator Mixing for Preparation of High-Solids Milk Protein-Stabilised Oil-in-Water Emulsions with Different Protein:Fat Ratios. J. Food Eng..

[26] Hall S., Cooke M., El-Hamouz A., Kowalski A.J. (2011). Droplet Break-up by in-Line Silverson Rotor–Stator Mixer. Chem. Eng. Sci..

[27] O’Sullivan J.J., Schmidmeier C., Drapala K.P., O’Mahony J.A., Kelly A.L. (2017). Monitoring of Pilot-Scale Induction Processes for Dairy Powders Using Inline and Offline Approaches. J. Food Eng..

[28] Van Der Schaaf U.S., Karbstein H.P. (2018). Fabrication of Nanoemulsions by Rotor-Stator Emulsification. Nanoemulsions.

[29] Schuch A., Deiters P., Henne J., Köhler K., Schuchmann H.P. (2013). Production of W/O/W (Water-in-Oil-in-Water) Multiple Emulsions: Droplet Breakup and Release of Water. J. Colloid Interface Sci..

[30] Pacek A.W., Hall S., Cooke M., Kowalski A.J., Tadros T.F. (2013). Emulsification in Rotor–Stator Mixers. Emulsion Formation and Stability.

[31] Rueger P.E., Calabrese R.V. (2013). Dispersion of Water into Oil in a Rotor–Stator Mixer. Part 2: Effect of Phase Fraction. Chem. Eng. Res. Des..

[32] Scholz P., Keck C.M. (2015). Nanoemulsions Produced by Rotor–Stator High Speed Stirring. Int. J. Pharm..

[33] Alonso-Miravalles L. (2020). Physicochemical Characterisation of Plant-Based Protein Ingredients for the Development of Infant Nutritional Formulations. Ph.D. Thesis.

[34] Malterre N., Bot F., O’Mahony J.A. (2023). Formulation and Physical Stability of High Total Solids Lentil Protein-Stabilised Emulsions for Use in Plant Protein-Based Young Child Formulae. Foods.

[35] De Figueiredo Furtado G., da Silva Carvalho A.G., Hubinger M.D. (2021). Model Infant Formulas: Influence of Types of Whey Proteins and Oil Composition on Emulsion and Powder Properties. J. Food Eng..

[36] Grasso N., Roos Y.H., Crowley S.V., Arendt E.K., O’Mahony J.A. (2021). Composition and Physicochemical Properties of Commercial Plant-Based Block-Style Products as Alternatives to Cheese. Future Foods.

[37] Laemmli U.K. (1970). Cleavage of Structural Proteins during the Assembly of the Head of Bacteriophage T4. Nature.

[38] Nakai S. (2003). Measurement of Protein Hydrophobicity. Curr. Protoc. Food Anal. Chem..

[39] Goulding D.A., O’Regan J., Bovetto L., O’Brien N.M., O’Mahony J.A. (2021). Influence of Thermal Processing on the Physicochemical Properties of Bovine Lactoferrin. Int. Dairy J..

[40] Vogelsang-O’Dwyer M., Sahin A.W., Bot F., O’Mahony J.A., Bez J., Arendt E.K., Zannini E. (2023). Enzymatic Hydrolysis of Lentil Protein Concentrate for Modification of Physicochemical and Techno-Functional Properties. Eur. Food Res. Technol..

[41] Alonso-Miravalles L., Jeske S., Bez J., Detzel A., Busch M., Krueger M., Wriessnegger C.L., O’Mahony J.A., Zannini E., Arendt E.K. (2019). Membrane Filtration and Isoelectric Precipitation Technological Approaches for the Preparation of Novel, Functional and Sustainable Protein Isolate from Lentils. Eur. Food Res. Technol..

[42] Pacek A.W., Ding P., Utomo A.T. (2007). Effect of Energy Density, pH and Temperature on de-Aggregation in Nano-Particles/Water Suspensions in High Shear Mixer. Powder Technol..

[43] Ding P., Orwa M.G., Pacek A.W. (2009). De-Agglomeration of Hydrophobic and Hydrophilic Silica Nano-Powders in a High Shear Mixer. Powder Technol..

[44] Dokoumetzidis A., Macheras P. (2006). A Century of Dissolution Research: From Noyes and Whitney to the Biopharmaceutics Classification System. Int. J. Pharm..

[45] Noyes A.A., Whitney W.R. (1897). The rate of solution of solid substances in their own solutions. J. Am. Chem. Soc..

[46] Padron G.A., Özcan-Taşkın N.G. (2018). Particle De-Agglomeration with an in-Line Rotor-Stator Mixer at Different Solids Loadings and Viscosities. Chem. Eng. Res. Des..

[47] Dikeman C.L., Fahey G.C. (2006). Viscosity as Related to Dietary Fiber: A Review. Crit. Rev. Food Sci. Nutr..

[48] Pereira R.N., Rodrigues R.M., Ramos Ó.L., Xavier Malcata F., Teixeira J.A., Vicente A.A. (2016). Production of Whey Protein-Based Aggregates Under Ohmic Heating. Food Bioprocess. Technol..

[49] Schuck P., Dolivet A., Jeantet R. (2012). Analytical Methods for Food and Dairy Powders.

[50] Melchior S., Moretton M., Calligaris S., Manzocco L., Nicoli M.C. (2022). High Pressure Homogenization Shapes the Techno-Functionalities and Digestibility of Pea Proteins. Food Bioprod. Process..

[51] Yang J., Liu G., Zeng H., Chen L. (2018). Effects of High Pressure Homogenization on Faba Bean Protein Aggregation in Relation to Solubility and Interfacial Properties. Food Hydrocoll..

[52] Yu S., Liu J.-J., Yun E.J., Kwak S., Kim K.H., Jin Y.-S. (2018). Production of a Human Milk Oligosaccharide 2′-Fucosyllactose by Metabolically Engineered Saccharomyces Cerevisiae. Microb. Cell Fact..

[53] Song X., Zhou C., Fu F., Chen Z., Wu Q. (2013). Effect of High-Pressure Homogenization on Particle Size and Film Properties of Soy Protein Isolate. Ind. Crops Prod..

[54] Bader S., Bez J., Eisner P. (2011). Can Protein Functionalities Be Enhanced by High-Pressure Homogenization?—A Study on Functional Properties of Lupin Proteins. Procedia Food Sci..

[55] Jarpa-Parra M., Bamdad F., Tian Z., Zeng H., Temelli F., Chen L. (2015). Impact of pH on Molecular Structure and Surface Properties of Lentil Legumin-like Protein and Its Application as Foam Stabilizer. Colloids Surf. B Biointerfaces.

[56] Barbana C., Boye J.I. (2013). In Vitro Protein Digestibility and Physico-Chemical Properties of Flours and Protein Concentrates from Two Varieties of Lentil (*Lens culinaris*). Food Funct..

[57] Joshi M., Adhikari B., Aldred P., Panozzo J.F., Kasapis S., Barrow C.J. (2012). Interfacial and Emulsifying Properties of Lentil Protein Isolate. Food Chem..

[58] Xu Y., Wang G., Wang X., Yu J., Wang J., Zhang Z., Li R. (2018). Effects of Homogenization on the Molecular Flexibility and Emulsifying Properties of Soy Protein Isolate. Food Sci. Biotechnol..

[59] Crowley S.V., Kelly A.L., O’Mahony J.A., Lucey J.A. (2019). Colloidal Properties of Protein Complexes Formed in β-Casein Concentrate Solutions as Influenced by Heating and Cooling in the Presence of Different Solutes. Colloids Surf. B Biointerfaces.

[60] Moll P., Salminen H., Schmitt C., Weiss J. (2021). Impact of Microfluidization on Colloidal Properties of Insoluble Pea Protein Fractions. Eur. Food Res. Technol..

